# Integrating Network Pharmacology and Experimental Validation: Mechanistic Study of the Anti-*Porphyromonas gingivalis* and Anti-Inflammatory Effects of *Berberis hemsleyana* Ahrendt Extract

**DOI:** 10.3390/plants15010115

**Published:** 2025-12-31

**Authors:** Guibin Yang, Mingan Gui, Hai Dong, Dongzhi Zhuoma, Xuehuan Li, Tai Shen, Hao Guo, Ruiying Yuan, Le Li

**Affiliations:** Department of Medicament, College of Medicine, Xizang University, Lhasa 850001, China; yangguibin0910@163.com (G.Y.);

**Keywords:** *Berberis hemsleyana* Ahrendt, antibacterial mechanism, anti-inflammatory, NF-κB pathway, network pharmacology

## Abstract

Anti-*Porphyromonas gingivalis* mechanisms of *Berberis hemsleyana* bark extract remain to be elucidated, and the anti-inflammatory activity of its n-butanol fraction (BNB) in RAW264.7 cells—mediated through suppression of the NF-κB pathway—require further validation. The minimum inhibitory concentration (MIC) and minimum bactericidal concentration (MBC) of the crude extract from *B. hemsleyana* were determined against *Candida albicans*, *Escherichia coli*, *Porphyromonas gingivalis*, *Staphylococcus aureus* and *Streptococcus mutans*. Scanning electron microscopy (SEM) and bacterial protein leakage assays were used to evaluate its antibacterial activity against *P. gingivalis*. High-performance liquid chromatography-mass spectrometry (LC-MS) was applied to analyze the ethanol extract of *B. hemsleyana* bark, leading to the screening of 47 compounds. The antibacterial mechanisms of the compounds were predicted through Network Pharmacology analysis and Molecular docking. Anti-inflammatory activity mediated via the NF-κB pathway was verified using an LPS-induced RAW264.7 cell inflammatory model. Specifically, the BNB showed a significant antibacterial effect on *P. gingivalis*. Meanwhile, it was confirmed that this fraction damaged the bacterial cell membrane structure, leading to the leakage of intracellular proteins in bacteria and thus impairing their infectivity. Network pharmacology analysis and molecular docking results indicated that *B. hemsleyana* bark’s biologically active compounds (Calenduloside E, Limonin, Acanthoside B, Dihydroberberine) antibacterial activity by regulating cytokines and cell apoptosis, thereby coordinating the body’s microbial homeostasis and inflammation. Additionally, BNB significantly reduced the secretion of the inflammatory cytokines IL-1β, TNF-α and IL-6 in vitro via the NF-κB pathway. The crude extract from the bark of *B. hemsleyana* has antibacterial and Anti-inflammatory activity. The n-butanol fraction showed a significant antibacterial effect on *P. gingivalis*.

## 1. Introduction

*Berberis hemsleyana* Ahrendt is distributed in Tibet, China, and grows in rock crevices at altitudes of 3660 to 4400 m. It has been traditionally used to treat dysentery with red mucus, jaundice, sore throat, conjunctivitis, and traumatic injuries in the Tibet region. It was revealed that *Berberis* genus plants possess pharmacological effects such as anti-inflammatory [1] and antioxidant properties [2]; furthermore, *Berberis* vulgaris and its constituent berberine reduce the production of inflammatory cytokines, such as interleukin-1β (IL-1β) and interleukin-6 (IL-6) [3].

To investigate the antimicrobial activity of the extract, five bacterial species were chosen. *Candida albicans*, *Porphyromonas gingivalis* and *Streptococcus mutans* are common pathogenic bacteria found in the oral cavity [4]. *S. aureusis* is a common pathogen associated with a variety of inflammatory diseases and systemic infections [5]. *P. gingivalis* is a prevalent oral pathogen and the core pathogenic bacterium responsible for periodontitis. Modern research has revealed that it is closely associated with systemic diseases such as cardiovascular diseases and rheumatoid arthritis [6]. *E. coli* resides in the human intestinal tract. Drug-resistant and pathogenic strains of *E. coli* can cause severe diarrhea and even result in death [7]. *C. albicans* is one of the commensal fungi in the human oral cavity, but it can transform into a pathogenic bacterium under specific conditions, and it is present on human skin and the reproductive tract. When the immune system is weakened, *C. albicans* invades and damages the skin and mucous membranes, leading to keratinization, ulcers, and erythema [8]. *S. mutans* is widely recognized as a key oral microorganism responsible for progressive tooth destruction, serving as the primary pathogenic agent in the development of dental caries [9].

Macrophages are key cells in the immune system involved in inflammatory responses. When bacterial lipopolysaccharide (LPS) triggers inflammation, macrophages are activated and express inducible nitric oxide synthase (iNOS), which catalyzes the production of nitric oxide (NO) from arginine. iNOS is closely associated with the inflammatory pathway NF-κB, an important pathway mediating nonspecific host defense mechanisms [10,11]. Activation of the TLR4 receptor on RAW264.7 cells by LPS leads to the activation of the NF-κB signaling pathway, accompanied by increased levels of phosphorylated IκB (p-IκB) and phosphorylated P65 (p-P65) within the cells. This ultimately results in elevated levels of inflammatory factors IL-1β and IL-6, contributing to the inflammatory response [12].

Screening for the bacteria that show higher susceptibility to the *B. hemsleyana* extract, and linking these strains to their respective diseases, serves as a basis for future research aimed at developing *B. hemsleyana* as a therapeutic agent for those diseases. This study will investigate the antibacterial capacity of *B. hemsleyana* and find the most antimicrobial solvent fraction for anti-inflammatory activity evaluation, while utilizing network pharmacology to predict the antibacterial mechanisms.

## 2. Materials and Methods

### 2.1. Chemicals and Reagents

*C. albicans* (GDMCC NO. 2.178), *E. coli* (GDMCC NO. 1.1299), *P. gingivalis* (GDMCC NO. 1.1464), *S. mutans* (GDMCC NO. 1.530) and *S. aureus* (GDMCC NO. 1.1348) were purchased from the Guangdong Microbial Culture Collection Center (GDMCC). RAW264.7 cells were cultured in a specialized medium obtained from Procell Life Science & Technology Co., Ltd. (Wuhan, China). The positive control drug (Metronidazole) was a commercially available tablet product that was procured from Xinyi Pharmaceutical Co., Ltd. (Shanghai, China). Ethanol, petroleum ether, ethyl acetate, and n-butanol were procured from Zhiyuan Chemical Reagent Co., Ltd. (Tianjin, China). Defibrinated sheep blood (Sterile, JS26086) was purchased from Henan Jushi Biotechnology Co., Ltd. Luria-Bertani broth medium (HB0128), tryptic soy agar medium (HB0177, TSA), tryptic soy broth medium (HB4114, TSB), and modified Sabouraud agar medium (HB0301) were obtained from Qingdao Haibo Biotechnology Co., Ltd. (Qingdao, China). The Coomassie Brilliant Blue staining solution for protein detection was procured from Beyotime Biotechnology Co., Ltd. (Shanghai, China). Radioimmunoprecipitation lysis buffer (RIPA), phenylmethanesulfonyl fluoride (PMSF), MTT cell proliferation and cytotoxicity assay kit and BCA protein assay kits were purchased from Solaibo Technology Co., Ltd. (Beijing, China). Primary antibodies, including mouse/rabbit TLR4 (AF7017), anti-P-P65 (AF2006), anti-P65 (BF8005), anti-P-IκB-α (AF2002), anti-IκB-α (AF5002), and B-actin, were procured from Affinity Biopharmaceutical Co., Ltd. (Nanjing, China). Horseradish peroxidase-conjugated goat immunoglobulin G (IgG) (ab150113) and rabbit anti-mouse IgG (ab150077) secondary antibodies were purchased from Abcam Plc. (Cambridge, UK).

### 2.2. Databases and Software

Databases and Software see Table 1.

### 2.3. Extraction of B. hemsleyana Barks

*B. hemsleyana* was collected from the surrounding areas of Lhasa. It was identified by Professor Zhuoma Dongzhi from the College of Medicine, Xizang University. Accurately weigh 200 g of *B. hemsleyana* barks, add 1600 mL of 70% ethanol, soak for 1 h, then perform ultrasonic extraction for 40 min three times. The extract was dried and then stored at 4 °C. A total of 20 g of the extract was suspended in 100 mL of water, and then sequentially extracted three times with equal volumes of petroleum ether, ethyl acetate, and n-butanol were dried and then stored at 4 °C. This extraction method adopts the commonly used extraction techniques in natural product research and selects ethanol as the solvent, with reference to Venanzoni et al. [13].

### 2.4. LC-MS Analysis of B. hemsleyana Extract

The experimental method refers to [14], and the detection conditions were determined after debugging based on the actual equipment and samples. Take the extract, add steel beads, place it in a grinder, and grind 6 times until it becomes a powder. Accurately weigh 50 mg of the powder, add 500 μL of 75% methanol-water solution (100 mg/mL), perform ultrasonic treatment for 30 min, then centrifuge at 17,000× *g* for 10 min. Collect the supernatant into a sample vial and load it onto the instrument for analysis. LC-MS conditions: Chromatographic Column (ACQUITY UPLC HSS T3 1.8 μm, 2.1 × 100 mm); Phase A: 0.1% formic acid in water; Phase B: 0.1% formic acid in acetonitrile. Flow rate: 0.4 mL/min; temperature: 40 °C; injection volume: 4 μL. 0–1.5 min, A95%; 2.5 min, A90%; 14 min, A60%; 24 min, A5%; 27 min, A5%; 27.01 min, A95%; 30 min, A95%.

Mass spectral data were acquired using an AB 5600 TQ-TOF mass spectrometer controlled by Analyst TF 1.7 software (AB Sciex), based on the Information-Dependent Acquisition (IDA) function. In each data acquisition cycle, molecular ions with the highest intensity (exceeding 100) were selected to collect the corresponding tandem mass spectrometry (MS/MS) data. The acquisition range was set to *m*/*z* 50–1200, and the collision energy was 30 eV. The parameters of the ESI ion source were set as follows: nebulizing gas pressure (GS1): 60 Psi; auxiliary gas pressure: 60 Psi; curtain gas pressure: 35 Psi; temperature: 550 °C; spray voltage: 5500 V (positive ion mode) or −4500 V (negative ion mode).

### 2.5. Agar Diffusion Method

The culture conditions for *C. albicans* are Sabouraud Dextrose Agar medium at 30 °C for 24 h. *E. coli* is cultured in LB broth medium at 37 °C for 24 h. *P. gingivalis* is cultured in LB nutrient broth medium with 10% defibrinated sheep blood under anaerobic conditions at 37 °C for 36 h. *S. mutans* and *S. aureus* are cultured in TSA nutrient broth medium at 37 °C for 24 h. Prepare various culture media according to the instructions, add them to Petri dishes, and allow the media to solidify before uniformly spreading 100 μL of bacterial suspension on the surface. Use an 8.0 mm Oxford cup to punch holes in the test plate, inject 80 μL of the test sample (at a concentration of 2 mg/mL) into each hole, and incubate for 24 h or 36 h. Conduct the experiment in triplicate, with 2 mg/mL Metronidazole as the positive control. After the incubation period, use a vernier caliper to measure and record the diameter of the inhibition circle [15].

### 2.6. Minimum Inhibitory Concentration (MIC) and Minimum Bactericidal Concentration (MBC)

The sample was diluted to a concentration range of 0.25–100 mg/mL using the two-fold serial dilution method. Add 50 μL of the diluted bacterial/fungal suspension and 200 μL of the sample at different concentrations into a 96-well plate. *S. aureus* and *E. coli* were incubated at 37 °C for 24 h, *S. mutans* and *P. gingivalis* were incubated in an anaerobic gas pack for 36 h, and *C. albicans* was incubated at 30 °C for 36 h. Observe the wells that remain clear—the corresponding concentration is defined as the MIC of the sample against the respective microorganism. During the cultivation of anaerobic bacteria, we utilize an anaerobic culture bag with an anaerobic gas-generating sachet, placed in a microbiological incubator to create an oxygen-free and constant-temperature environment at 37 °C for 36 h.

Aspirate 50 μL of the liquid separately from the drug-negative control wells (clear) and the two wells subsequent to the MIC well in the 96-well plate, and spread it on agar plates, followed by incubation at 37 °C for 24 h. Quantitative analysis was performed using the colony-forming unit (CFU) counting method. The MBC is defined as the lowest drug concentration at which the viable bacterial count after drug treatment is reduced to a threshold of 0.1% of the initial inoculum [16].

### 2.7. Membrane Permeability Assay

The activated *P. gingivalis* was cultured in a constant temperature shaker (37 °C, 220 rpm) for 6 h. Bacteria in the logarithmic growth phase were centrifuged at 5000 rpm for 5 min, and the supernatant was discarded. The bacterial pellet was resuspended in an equal volume of phosphate-buffered saline (PBS) to a final concentration of 1 × 10^8^ CFU/mL. An equal volume of the *B. hemsleyana* n-butanol extract solution (BNB) was added, adjusting the final concentrations to 1/4 MIC (62.5 μg/mL), 1/2 MIC (125 μg/mL), MIC (250 μg/mL), 2 MIC (500 μg/mL), 4 MIC (1000 μg/mL), as well as Metronidazole (the positive control). The mixture was incubated in a shaker at 37 °C for 2 h and 4 h. The blank control group was treated with an equal volume of PBS. After the bacterial suspension was filtered through a 0.22 μm filter membrane, 2 μL of the filtrate was collected to determine the protein concentration using a BCA protein assay kit, with reference to the standard curve [17].

### 2.8. Biological Scanning Electron Microscopy (SEM)

Following the method of Chen et al. [18], BNB solution at the MIC concentration or PBS (control) was added to a suspension of *P. gingivalis* in the logarithmic growth phase (5 × 10^8^ CFU/mL). After 6 h of incubation, the bacteria were centrifuged using a high-speed centrifuge (8000 rd/min). The pelleted bacteria were resuspended and washed three times with PBS. The samples were fixed with 2.5% (*v*/*v*) glutaraldehyde in 0.1 M sodium cacodylate buffer, pH 7.2, 4 °C, for 4 h. Subsequently, they were dehydrated through a graded ethanol series (30%, 50%, 70%, 80%, 90%, and 100%) every time for 15 min, dried at the critical point with hexamethyldisilazane (HMDS) (BAL-TEC, CPD 030), and coated with gold. The sample morphology was observed using a Hitachi SU8010 ultra-high resolution field emission scanning electron microscope. Three samples were observed per group.

### 2.9. Prediction of Active Components and Disease Targets

The chemical components in *B. hemsleyana* extract were input into the PubChem database to obtain the SMILES format of compound structures. These structures were then submitted to the SwissTargetPrediction database to query the gene targets of drug action. Meanwhile, the gene targets collected from the TCMSP and ETCM databases were merged, and their names were standardized and unified using the UniProt database. Finally, the active component-target network diagram was constructed with Cytoscape 3.10.1 software.

In the UniProt and Gene Cards databases, using “antibacterial” and “antimicrobial” as keywords and restricting the species to Homo sapiens (human), human gene targets related to antibacterial and antimicrobial functions were obtained. A Venn diagram was drawn using Venny 2.1.0 [19].

### 2.10. Construction of Protein–Protein Interaction Network and Screening of Core Targets

The intersection 28 targets obtained in Section 2.9 were input into the STRING database for protein–protein interaction network analysis. Utilize the database within the website to interpret the connections of the target protein and screen for proteins with a node count > 10. The processed data were imported into Cytoscape 3.10.1 software, and target screening was performed based on degree values and other criteria, with simultaneous construction of the network diagram [19].

### 2.11. KEGG Pathway Analysis and GO Enrichment Analysis

The intersection genes were imported into the online Metascape platform to perform KEGG pathway enrichment analysis and GO functional analysis. Locate annotation tool Metascape. Upload the sequence file, select the corresponding species, and submit the task. The KEGG analysis results will identify the key mechanisms or pathways corresponding to the crucial proteins and perform enrichment analysis on the proteins associated with each pathway. The steps for GO analysis are consistent with this: it is necessary to select three different analysis modes, biological process (BP), molecular function (MF) and cellular component (CC) and then record the enrichment results. The obtained data were organized and input into the XiaoeBio website for visualization processing [19].

### 2.12. Molecular Docking

Major active compounds in SDF format were downloaded from the PubChem database, and the 3D structures of core target proteins were downloaded from the UniProt database. The PyMOL 2.5.0 software was used to remove water molecules and heteromolecules from the proteins. Docking was performed on the CB-DOCK2 platform. After docking, the binding energy was recorded, and PyMOL 2.5.0 software was used for molecular docking visualization, with the 3D structures output [20].

### 2.13. Cell Culture and Viability Assay

RAW264.7 cells were cultured in Dulbecco’s Modified Eagle Medium (DMEM) with NEAA supplemented with 10% FBS and 1% penicillin/streptomycin (P/S). The cells were maintained at 37 °C in a 5% CO_2_ environment, with sub-culturing performed every 1 day. RAW264.7 cells were cultured in 96-well plates at a density of 2 × 10^5^ cells/mL, with 100 μL of cell suspension added to each well. The plates were then incubated for 24 h. The cells were exposed to BNB either alone or at varying concentrations (0, 12.5, 25, 50, 100, 200 μg/mL). The plates were returned to the incubator for another 24 h. Subsequently, a solution of 3-[4,5-dimethylthiazol-2-yl]-2,5-diphenyltetrazolium bromide (MTT, 2.5 mg/mL, 50 μL) was added to each well. After a 4 h incubation, formazan crystals were dissolved in Dimethyl sulfoxide (DMSO), and the optical density (OD) was measured at a wavelength of 570 nm. The initial OD was recorded at the time of compound addition [21].

### 2.14. Measurement of Inflammatory Cytokines

The cells were exposed to BNB either alone or at varying concentrations (12.5, 25 and 50 μg/mL) for 24 h. Additionally, the cells received an extra treatment with LPS (1 μg/mL) for an additional 12 h. The concentration levels of the inflammatory factors IL-6 and IL-1β were confirmed successful model group establishment using reagent kits [21].

### 2.15. Western Blot Analysis

Protein expression of relevant targets was detected by Western blot. Total protein was quantified using the BCA method. RAW264.7 cell lysates (containing protease inhibitors) were fully lysed, and the proteins were separated using an SDS-PAGE system. Subsequently, the proteins were transferred onto a PVDF membrane via the wet transfer method. The membrane was then blocked with 5% skimmed milk in TBST for 1 h at room temperature. Following blocking, the membrane was incubated with a primary antibody (1:1000) at 4 °C overnight. Then the membrane was washed with TBST three times, followed by incubation with an HRP-conjugated secondary antibody (1:5000) for 1 h at room temperature. After another three washes with TBST, the bands were visualized using an ECL chemiluminescence substrate. Band intensity was analyzed using ImageJ software. β-actin was used as the internal control, and the relative expression level of the target proteins was calculated as the ratio of the target protein band intensity to that of β-actin. Finally, GraphPad software was used to process the data [22].

Meanwhile, the proteins from the *P. gingivalis* experimental group were separated using the SDS-PAGE system, and the resulting gel was stained with Coomassie Brilliant Blue for observation.

### 2.16. Statistical Methods

Statistical analysis was performed using SPSS 27.0 and GraphPad 9.5.0 statistical software. The antibacterial experiment was conducted with three independent replicates (*n* = 3). Data are presented as mean ± standard deviation. Comparisons between groups were analyzed by an independent samples *t*-test, and a *p*-value < 0.05 was considered statistically significant.

## 3. Results

### 3.1. Component Analysis of B. hemsleyana Extract

Total ion currents in positive and negative modes were subjected to comparative analysis of mass spectrometry data and identified using mass spectrometry databases. A total of 1083 compounds were detected, and 47 compounds were screened out using signal intensity, content proportion, and ppm < 5 as criteria, as shown in Table 2 and Figure S1.

### 3.2. Screening of Antibacterial Activity of B. hemsleyana Extract

**Figure 1:**
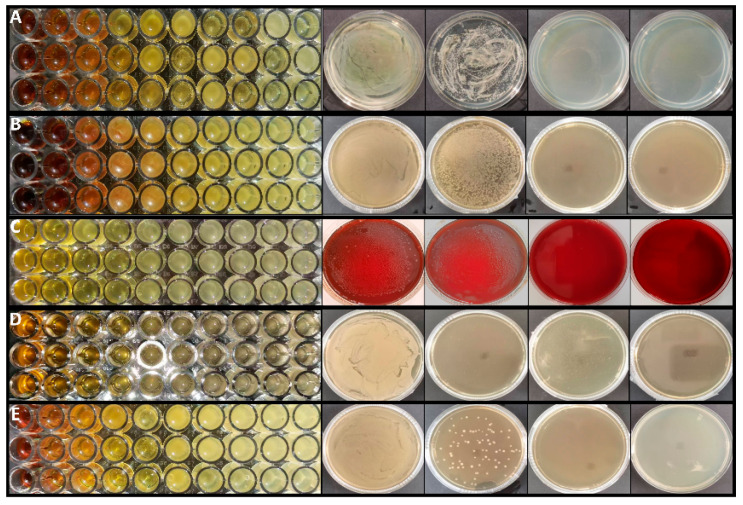
Determination of the antibacterial and bactericidal effects of extract of *B. hemsleyana.* The MIC and MBC of the (**A**) *C. albicans*, (**B**) *E. coli*, (**C**) *P. gingivalis*, (**D**) *S. aureus*, (**E**) *S. mutans* by extract of *B. hemsleyana*.

The inhibitory activity of the ethanol extract of *B. hemsleyana* against common pathogenic bacteria, including *C. albicans*, *E. coli*, *P. gingivalis*, *S. aureus* and *S. mutans*, was investigated to explore the antibacterial activity of *B. hemsleyana* extract. It was found that *B. hemsleyana* extract exhibited significant inhibitory effects on *P. gingivalis* and *S. aureus*, as shown in Figure 1 and Table 3.

**Figure 2:**
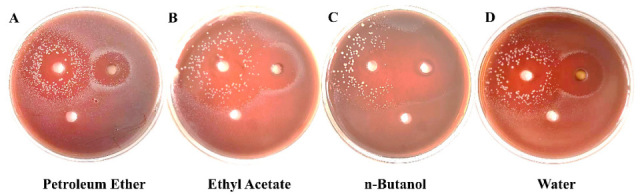
The influence of *B. hemsleyana* (**A**) petroleum ether (BPE), (**B**) ethyl acetate (BEA), (**C**) n-butanol (BNB), (**D**) water (BW) components on the antibacterial zone.

The anti-*P. gingivalis* activity of each extract fraction of *B. hemsleyana* is shown in Figure 2, Table 4 and Table 5. The results indicate that compared with the positive control drug Metronidazole, the petroleum ether, ethyl acetate, and n-butanol fractions of *B. hemsleyana* all exhibit certain inhibitory activity. Among them, the BNB showed the strongest inhibitory activity against *P. gingivalis*.

### 3.3. BNB Damages the Bacterial Cell Membrane of P. gingivalis

**Figure 3:**
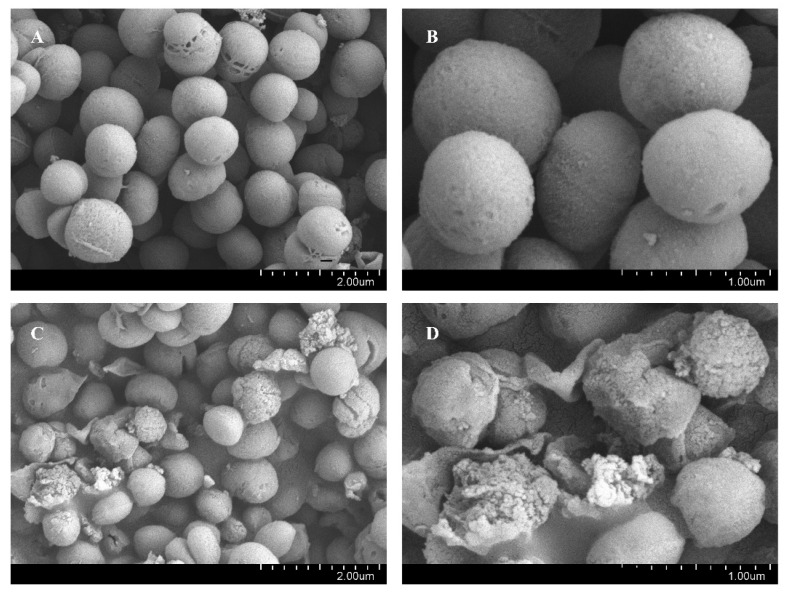
Scanning electron microscope results of BNB treatment of *P. gingivalis* for 6 h. (**A**,**B**) Control group (**C**,**D**) and BNB (0.25 mg/mL), 20,000× and 50,000×.

**Figure 4:**
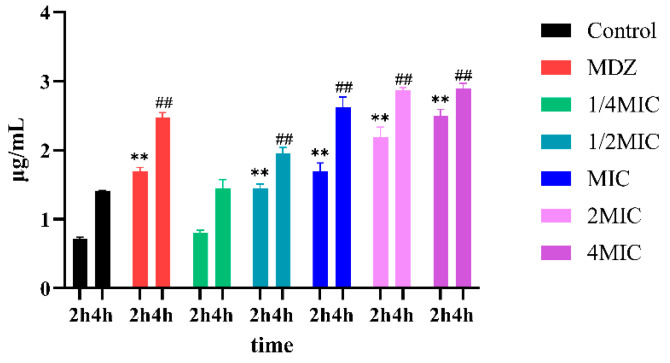
Intracellular protein leakage of *P. gingivalis*. (MDZ: Metronidazole 1 mg/mL, MIC: BNB 0.25 mg/mL). Results are mean ± SD (n = 3). ** *p* < 0.01 vs. control group at 2 h; ## *p* < 0.01 vs. control group at 4 h.

SEM was used to observe the effects of BNB on the morphology and structure of *P. gingivalis*. The results showed that BNB induced significant changes in the morphology of *P. gingivalis* in Figure 3. The untreated *P. gingivalis* exhibited a spherical cell morphology with an intact bacterial membrane, while the *P. gingivalis* treated with the BNB showed damaged bacterial membranes, along with rough, wrinkled, and irregular spherical surfaces. Meanwhile, in Figure 4, the effect of BNB on the cell membrane permeability of *P. gingivalis* indicated that the administration group had increased protein leakage, suggesting that the cell membrane was damaged. Moreover, the damage became more severe with the extension of administration time, thus demonstrating that BNB exhibited significant antibacterial activity. Concurrently, we observed via SDS-PAGE and Coomassie Blue staining that BNB may disrupt bacterial protein synthesis. Compared with the normal bacteria group, the protein bands in the BNB-treated group were fainter, a result that was also observed with the positive control drug, Metronidazole (Figure S2).

### 3.4. Collection of Action Targets of B. hemsleyana Extract

**Figure 5:**
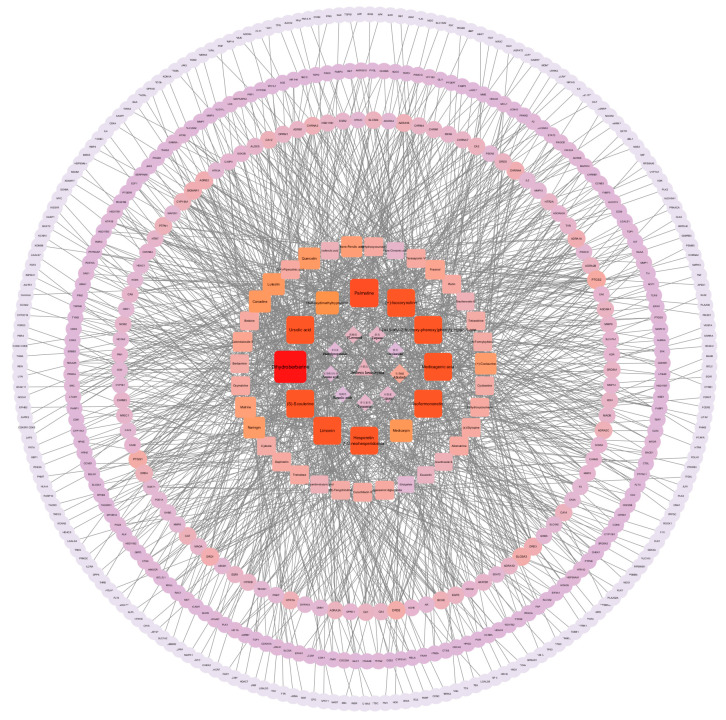
PPI network map of *B. hemsleyana* bark targets.

Through target screening and integration of the 47 identified compounds, a total of 538 gene targets were obtained in Figure 5. Based on these, a “drug-component classification-compound-gene target” network was constructed, consisting of 596 nodes and 1321 edges, indicating that *B. hemsleyana* extract exhibits multi-component and multi-target characteristics. The network structure revealed that node size and color intensity are positively correlated with the number of associated genes, visually reflecting the differential importance of various compounds and their classifications in target regulation.

### 3.5. Screening of Key Antibacterial Targets of B. hemsleyana

A total of 250 main antibacterial and antimicrobial targets of Homo sapiens were collected from the UniProt and Gene Cards databases. The action targets of the components of *B. hemsleyana* and human antibacterial gene targets were input into the Venny 2.1 website, resulting in 28 intersection genes, accounting for 3.7% of the union targets. The common targets of the drug and antibacterial activity were entered into the STRING database, and the associated genes were expanded to 54 genes. A protein–protein interaction (PPI) network of the antibacterial targets of *B. hemsleyana* was constructed, and the data were imported into Cytoscape 3.10.1 software for mapping. The PPI network diagram consists of 54 nodes and 746 edges, with an adjusted *p* < 1.0 × 10^−16^, an average degree value of 27.6, and a clustering credibility value of 0.829. Topological property analysis was performed using CytoNCA, and 14 core targets were selected based on the criteria of degree > 35, betweenness unDir > 30.9, and closeness centrality > 0.7. Meanwhile, the STRING database indicated that CASP7, BIRC5, CDK9, STAT1, and RELA are intersection genes significantly associated with the core targets. Therefore, a total of 19 genes were obtained. After excluding the genes previously expanded using STRING from the 19 genes, 14 gene targets were finally identified, along with their corresponding compounds.

**Figure 6:**
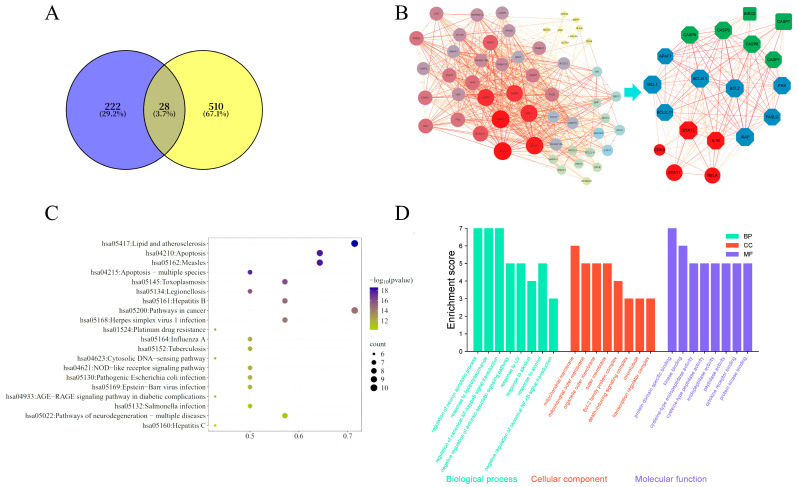
Network pharmacology analysis of the active effects of *B. hemsleyana* extract. (**A**) Intersection of drug targets and disease targets. (**B**) Core target PPI network diagram. (**C**) KEGG pathway enrichment analysis. (**D**) GO functional enrichment analysis.

The integration of 250 human antibacterial targets with the predicted targets of *B. hemsleyana* extract components revealed 28 initial intersection genes. Protein–protein interaction (PPI) network analysis expanded this set to 54 genes, forming a highly interconnected network (746 edges) with significant reliability (*p* < 1.0 × 10^−16^). Topological analysis identified 14 core targets based on centrality metrics, and five key intersection genes (CASP7, BIRC5, CDK9, STAT1, RELA) were highlighted as significantly associated with core network functions, including inflammatory bowel disease and apoptosis. The final selection process yielded 14 high-confidence gene targets, which are proposed as the primary mediators of the antibacterial effects of *B. hemsleyana* extract, as shown in Figure 6A,B and Table 6.

### 3.6. Biological Function and Pathway Analysis

Based on KEGG and GO enrichment analyses, the potential antibacterial targets of *B. hemsleyana* extract were significantly enriched in 20 KEGG pathways, including apoptosis, primarily involving infectious diseases, immune regulation, and apoptotic processes. GO classification revealed enrichment in biological processes such as neuronal apoptosis and NF-κB signaling regulation, cellular components including mitochondrial and plasma membranes, and molecular functions such as kinase binding and cysteine-type endopeptidase activity. These results suggest that it may exert its multi-target antibacterial effects by regulating pathways related to apoptosis, immune-inflammatory responses, and pathogen infection, as shown in Figure 6C,D.

#### Molecular Docking Validation

**Figure 7:**
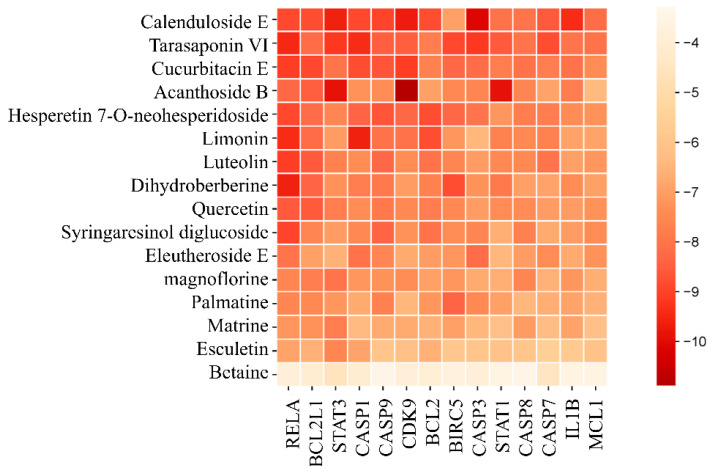
Heatmap of binding energies between key targets and active compounds.

**Figure 8:**
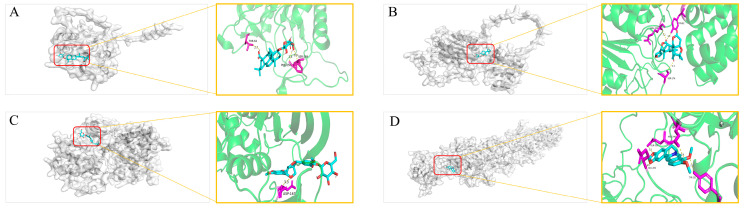
Visualization analysis of integration. (**A**) Calenduloside E—CASP3, (**B**) Limonin—CASP1, (**C**) Acanthoside B—CDK9, (**D**) Dihydroberberine—RELA.

Based on the values of relevant targets degree, betweenness centrality, and closeness centrality, the frequency of active components, and the relevant literature, 17 active components including Calenduloside E, Tarasaponin VI, Cucurbitacin E, Echinacoside B, Hesperetin 7-O-neohesperidoside, Citric Acid, Luteolin, Dihydroberberine, Quercetin, Syringaresinol, Liriodendrin, Magnoflorine, Berbamine, Matrine, Aesculetin, and Betaine—were selected for molecular docking with 14 core targets (BCL2, BCL2L1, BIRC5, CASP1, CASP3, CASP7, CASP8, CASP9, CDK9, IL1B, MCL1, RELA, STAT1, and STAT3) (Figure 7). The lower the binding energy between the small molecule ligand and the protein receptor, the more stable the bound molecules, indicating a higher probability of intermolecular interactions. The docking results with the highest binding affinity were selected for visualization, in Figure 8 which are as follows: A. Calenduloside E—CASP3’s binding energy is −10 kcal·mol^−1^, B. Limonin—CASP1’s binding energy is −9.6 kcal·mol^−1^, C. Acanthoside B—CDK9’s binding energy is −10.9 kcal·mol^−1^, and D. Dihydroberberine—RELA’s binding energy is −9.6 kcal·mol^−1^. Then, the in vitro antibacterial activity of the four compounds was evaluated, and dihydroberberine demonstrated significant antibacterial activity (Figure S3).

### 3.7. BNB Reduces the Expression of Inflammatory Factors in LPS-Induced RAW264.7 Cell Inflammation

**Figure 9:**
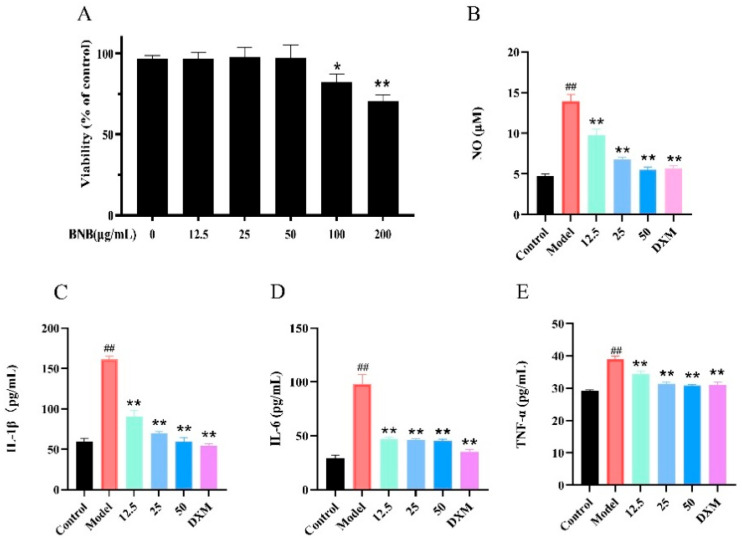
Effect of BNB on inflammation in LPS-induced RAW264.7 cells. (**A**) Effect of BNB on the viability of RAW264.7 cells. The levels of (**B**) NO, (**C**) IL-1β, (**D**) IL-6, (**E**) TNF-α content. Results are mean ± SD (*n* = 3). ## *p* < 0.01 vs. control group; * *p* < 0.05, ** *p* < 0.01 vs. model group.

Cell viability assays indicated that BNB exhibited low cytotoxicity at concentrations below 100 μg/mL. Therefore, concentrations of 12.5 μg/mL, 25 μg/mL, and 50 μg/mL were selected as the low, medium, and high dose groups, respectively (Figure 9A), and the effect of SMS on the release of NO and inflammatory factors (TNF-α, IL-1β and IL-6) was assessed. Treatment with BNB inhibited the levels of NO, TNF-α, IL-1β, and IL-6 compared with the LPS group in a dose-dependent manner. Taken together, these results indicate that they reduce the release of inflammatory factors in vitro. BNB significantly suppressed the LPS-induced secretion of NO, TNF-α, IL-1β, and IL-6 in RAW264.7 cells in a concentration-dependent manner, as shown in Figure 9B–E.

### 3.8. Effect of BNB on the Expression of NF-κB Pathway-Related Proteins in LPS-Induced RAW264.7 Cells

**Figure 10:**
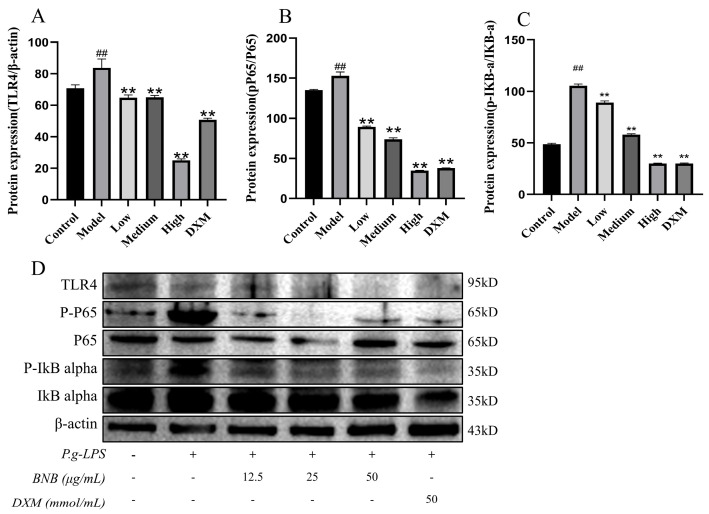
BNB inhibits the NF-κB pathway proteins in LPS-induced RAW264.7 cells. The expressions of (**A**) TLR4, (**B**) P-P65/P65, (**C**) P-IkB-α/IkB-α were detected by Western blot, (**D**) The proteins expression figure of NF-κB pathway was analyzed by Western blot. Results are mean ± SD (*n* = 3). ## *p* < 0.01 vs. control group. ** *p* < 0.01 vs. model group.

**Figure 11:**
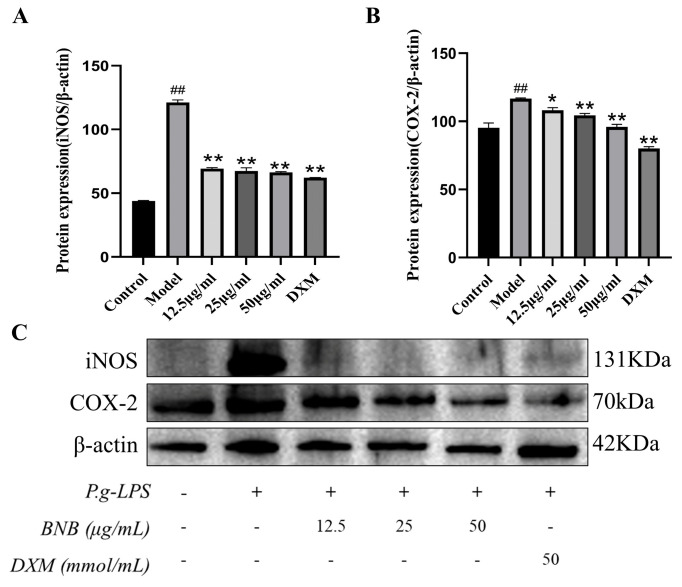
BNB inhibits the expression of COX-2 and iNOS. The expressions of (**A**) iNOS and (**B**) COX-2 were detected by Western blot, (**C**) The proteins expression figure of iNOS and COX-2 were analyzed by Western blot. Results are mean ± SD (*n* = 3). ## *p* < 0.01 vs. control group, * *p* < 0.05, ** *p* < 0.01 vs. model group.

To assess whether BNB regulates the level of inflammation through the NF-κB pathway, Western blot analysis demonstrated that the markedly elevated expression levels of TLR4, p-P65/P65, and p-IkB-α/IkB-α in the LPS group were dose-dependently reversed by BNB, as shown in Figure 10A–C, by inhibiting the NF-κB pathway. BNB significantly inhibits the levels of iNOS and COX-2 proteins in inflammatory RAW264.7 cells, which are mediated by the NF-κB pathway, as shown in Figure 11A,B.

## 4. Discussion

This study identified the compounds in the extract of *B. hemsleyana* via LC-MS. A total of 47 compounds have been identified, including alkaloids, flavonoids, triterpenoids, coumarins, and lignans. Meanwhile, from the perspective of antibacterial activity in vitro, the antibacterial effects were evaluated with five pathogenic microorganisms (*C. albicans*, *E. coli*, *P. gingivalis*, *S. aureus*, and *S. mutans*) and their approximate MICs and MBCs were preliminarily determined. Notably, the extract exhibited significant inhibitory activity against *P. gingivalis* (1.25 ± 0.05 mg/mL). Therefore, components of different polarities were used to test their inhibitory activity against *P. gingivalis*, and it was found that the BNB exerted remarkable inhibitory effects on *P. gingivalis*. SEM observations revealed that BNB achieves antibacterial effects by disrupting the biofilm of *P. gingivalis* after 6 h. The experiment on intracellular protein leakage of *P. gingivalis* showed that 1 mg/mL BNB damages the bacterial cell membrane, and its effect is comparable to that of Metronidazole. This suggests that the BNB has inhibitory and therapeutic potential against bacterial infections associated with periodontitis, which warrants further investigation [23].

Based on the compounds identified in the crude *B. hemsleyana* extract, network pharmacology was used to predict the mechanistic pathways through which *B. hemsleyana* extract has antibacterial effects on the human body. Key targets worthy of attention, such as BCL2, BIRC5, CDK9, IL1B, MCL1, and CASPs (caspase family), were identified. This indicates that *B. hemsleyana* extract coordinates host microbial homeostasis and inflammation by regulating cytokines and cell apoptosis, thereby exerting antibacterial and antimicrobial activities [24]. In the prediction of the anti-Legionellosis pathway, studies have shown that this type of bacteria can act on the host cell’s membrane to prevent macrophage phagocytosis, block host cell autophagy, and interfere with cell apoptosis, thereby enabling their reproduction and infection. In contrast, the GO analysis indicated that the targets of *B. hemsleyana* extract are associated with cellular substructures such as the organelle outer membrane, plasma membrane, and tubulin. This suggests that it may prevent Legionella from interfering with the cell membrane, which supports the prediction of its antibacterial activity [25]. Pathogenic *E. coli* infection is consistent with the favorable antibacterial activity of the crude extract against the *E. coli* observed in the antibacterial assay. Meanwhile, studies have shown that berberine, when combined with antibacterial agents, can enhance the in vitro antibacterial efficacy of drugs against animal-derived *E. coli* [26]. Molecular docking experiments were performed to visualize and score the binding affinity between key targets and active compounds [27]. The results revealed that Calenduloside E with CASP3, Citric Acid with CASP1, Acanthoside B with CDK9, and Dihydroberberine with RELA exhibited relatively low binding energies, indicating stable intermolecular interactions.

When bacterial invasion activates intracellular NF-κB/MAPK signaling pathways, CDK9 is rapidly recruited to the promoter regions of immune genes, initiating the transcription of TNF-α, IL-6, and IL-1β genes and guiding immune cell migration to the infection site [28]. Bacterial infections are often accompanied by tissue inflammation, and the NF-κB signaling pathway is the most typical inflammatory pathway [29]. By studying the inhibitory effect of BNB on LPS-induced inflammation in RAW264.7 cells, we found that BNB can suppress the expression of TLR4 membrane receptors and inhibit the phosphorylation of P65 and IkB-α proteins, thereby restraining the overactivation of the NF-κB pathway. This leads to a reduction in the expression of the inflammatory factors TNF-α, NO, IL-1β, IL-6, iNOS, and COX-2, ultimately alleviating inflammation.

In conclusion, the study predicted the network relationships among the active components and pathways of *B. hemsleyana* bark via network pharmacology and antibacterial experiments in vitro. Future studies will focus on its key targets and signaling pathways to further conduct in vivo and clinical research and systematically investigate the molecular mechanism underlying its antibacterial activity through in-depth molecular experiments.

## Figures and Tables

**Table 1:** Databases and software.

Database	Web Link
TCMSP (Traditional Chinese Medicine Systems Pharmacology Database and Analysis Platform)	https://www.tcmsp-e.com/
ETCM (The Encyclopedia of Traditional Chinese Medicine)	http://www.tcmip.cn/ETCM/
Swiss Target Prediction	https://swisstargetprediction.ch/
UniProt	https://www.uniprot.org/uniprotkb
Gene Cards	https://www.genecards.org/
STRING	https://cn.string-db.org/
Metascape	https://metascape.org/
PubChem	https://pubchem.ncbi.nlm.nih.gov/
Venny 2.1.0	https://bioinfogp.cnb.csic.es/tools/venny/index.html
Wei Sheng Xin Platform	http://www.bioinformatics.com.cn/
CB-DOCK2	https://cadd.labshare.cn/cb-dock2/php/index.php
MSDIAL version 4.6 software	http://prime.psc.riken.jp/
Cytoscape 3.10.1 software	https://cytoscape.org/
PyMOL 2.5.0 software	https://pymol.org/
ImageJ software	https://imagej.net/ij/
GraphPad Pism9.5 software	https://www.graphpad.com/

**Table 2:** LC-MS analysis for the identification of compounds in *B. hemsleyana* bark.

No.	Compound Name	CAS	m/z	ppm	Ion Mode	Class
1	Dihydroberberine	120834-89-1	338.1378	2.6	POS	Tyrosine alkaloids
2	(R)-Fangchinoline	33889-68-8	609.7138	3.4	POS	Tyrosine alkaloids
3	Berbamine	478-61-5	609.2938	3.4	POS	Tyrosine alkaloids
4	Oxymatrine	16837-52-8	265.19	4.2	POS	Lysine alkaloids
5	(+)-Coclaurine	2196-60-3	286.1429	3.2	POS	Tyrosine alkaloids
6	5-Methoxydimethyltryptamine	1019-45-0	219.1485	3.3	POS	Tryptophan alkaloids
7	4-Guanidinobutyric acid	463-00-3	146.0926	1	POS	Small peptides
8	D(+)-Pipecolinic acid	1723-00-8	130.0858	3.6	POS	Small peptides
9	Fraxinol	486-28-2	223.0595	2.9	POS	Coumarins
10	Matrine	519-02-8	249.1951	4.3	POS	Lysine alkaloids
11	Allomatrine	641-39-4	249.1951	4.3	POS	Lysine alkaloids
12	N-Formylcytisine	53007-06-0	219.1123	2.6	POS	Lysine alkaloids
13	Canadine	522-97-4	340.1531	3.7	POS	Tyrosine alkaloids
14	Betaine	107-43-7	118.0859	3.6	POS	Small peptides
15	Tetrandrine	518-34-3	623.3088	4.4	POS	Tyrosine alkaloids
16	Cycleanine	518-94-5	623.3088	4.4	POS	Tyrosine alkaloids
17	(±)-Stylopine	138791-29-4	324.1223	2.4	POS	Tyrosine alkaloids
18	6-Hydroxycoumarin	6093-68-1	161.0242	1.4	NEG	Coumarins
19	4-Hydroxycoumarin	1076-38-6	161.0242	1.4	NEG	Coumarins
20	trans-Ferulic acid	537-98-4	193.0503	1.5	NEG	Phenylpropanoids (C6-C3)
21	Isoferulic acid	25522-33-2	193.0503	1.5	NEG	Phenylpropanoids (C6-C3)
22	Esculetin	305-01-1	177.019	1.6	NEG	Coumarins
23	Daphnetin	486-35-1	177.019	1.6	NEG	Coumarins
24	Acanthoside B	7374-79-0	579.2072	1.8	NEG	Lignans
25	Eleutheroside E	573-44-4	741.2602	1.3	NEG	Lignans
26	Syringaresinol diglucoside	66791-77-3	741.2602	1.3	NEG	Lignans
27	Acanthoside D	96038-87-8	741.2602	1.3	NEG	Lignans
28	Liriodendrin	573-44-4	741.2602	1.3	NEG	Lignans
29	Trehalose	99-20-7	341.1081	2.3	NEG	Saccharides
30	Quercetin	117-39-5	303.0492	2.4	POS	Flavonoids
31	Rutin	153-18-4	609.1459	0.3	NEG	Flavonoids
32	Luteolin	491-70-3	285.0401	1.2	NEG	Flavonoids
33	Medicarpin	33983-40-3	271.0958	2.7	POS	Isoflavonoids
34	Ethylgallate	831-61-8	197.0452	1.6	NEG	Phenolic acids (C6-C1)
35	Cytisine	485-35-8	191.1174	2.7	POS	Nicotinic acid alkaloids
36	(+)-Isocorynoline	475-67-2	340.1546	2.3	NEG	Tyrosine alkaloids
37	Isomagnolone	155709-41-4	281.1177	2	NEG	Lignans
38	(S)-Scoulerine	6451-73-6	328.1537	1.9	POS	Tyrosine alkaloids
39	Naringin	10236-47-2	581.1844	3.6	POS	Flavonoids
40	Isoformononetin	486-63-5	269.0804	1.8	POS	Isoflavonoids
41	Hesperetin 7-O-neohesperidoside	13241-33-3	609.1811	2.2	NEG	Flavonoids
42	Ursolic acid	77-52-1	455.3526	1	NEG	Triterpenoids
43	Tarasaponin VI	59252-95-8	763.4262	1.5	NEG	Triterpenoids
44	Limonin	1180-71-8	471.2006	1.7	POS	Triterpenoids
45	Medicagenic acid	599-07-5	501.3213	1.7	NEG	Triterpenoids
46	Calenduloside E	26020-14-4	631.3846	0.9	NEG	Triterpenoids
47	Cucurbitacin E	18444-66-1	555.2939	4.4	NEG	Triterpenoids

**Table 3:** MIC and MBC of *B. hemsleyana* extract against five types of bacteria.

Strain	MIC (mg/mL)	MBC (mg/mL)
*C. albicans*	6.25 ± 0.05	12.00 ± 0.05
*E. coli*	25.00 ± 0.05	50.00 ± 0.05
*P. gingivalis*	1.25 ± 0.05	5.00 ± 0.05
*S. aureus*	2.50 ± 0.05	5.00 ± 0.05
*S. mutans*	6.25 ± 0.05	25.00 ± 0.05

**Table 4:** Inhibitory agar plate inhibition zone diameter table.

Extraction Solvent	*P. gingivalis* Inhibition Zone (mm)
Petroleum Ether	16.1 ± 0.30
Ethyl Acetate	22.07 ± 0.35
n-Butanol	36.2 ± 0.30
Water	26.97 ± 0.21

**Table 5:** MIC and MBC concentrations of various extracts of *B. hemsleyana* bark against *P. gingivalis*.

Extraction Solvent	MIC (mg/mL)	MBC (mg/mL)
Petroleum Ether	2.00 ± 0.01	8.00 ± 0.01
Ethyl Acetate	2.00 ± 0.01	4.00 ± 0.01
n-Butanol	0.25 ± 0.01	0.50 ± 0.01
Water	1.00 ± 0.01	2.00 ± 0.01

**Table 6:** Core antibacterial target of *B. hemsleyana* extract.

Gene	Corresponding Representative Active Components
*CASP7*	Luteolin
*CASP8*	Quercetin
*CASP9*	Quercetin
*CASP1*	Citric Acid
*CASP3*	Citric Acid, Calenduloside E
*BIRC5*	Palmatine, Quercetin, Luteolin
*STAT3*	Calenduloside E, Magnoflorine
*IL1B*	Quercetin
*STAT1*	Quercetin
*CDK9*	Citric Acid, Dihydroberberine
*RELA*	Betaine, Matrine, Quercetin
*BCL2*	Aesculetin
*MCL1*	Luteolin, Acanthopanaxoside B, Liriodendrin
*BCL2L1*	Calenduloside E, Tarasaponin VI

## Data Availability

The original contributions presented in this study are included in the article/Supplementary Material. Further inquiries can be directed to the corresponding author.

## Supplementary Materials

The following supporting information can be downloaded at: https://www.mdpi.com/article/10.3390/plants15010115/s1.
