# Supplementary material for: Integrating Network Pharmacology and Experimental Validation: Mechanistic Study of the Anti-Porphyromonas gingivalis and Anti-Inflammatory Effects of Berberis hemsleyana Ahrendt Extract
Source: Plants (Basel). 2025 Dec 31;15(1):115. doi: 10.3390/plants15010115 (PMC12787952; doi:10.3390/plants15010115)
Supplement: Supplementary file 1 [file plants-15-00115-s001.zip › plants-4039487-supplementary.pdf]

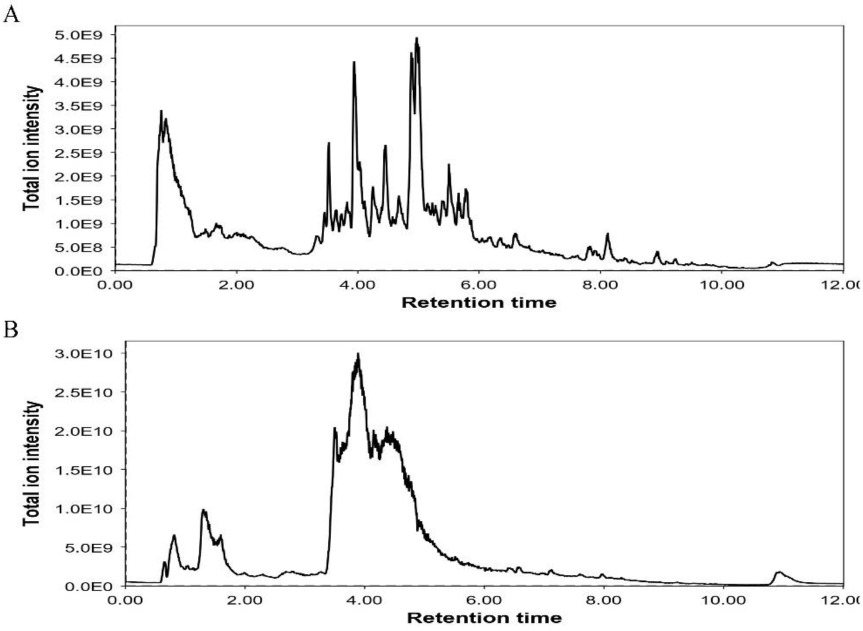

Figure S1. *Berberis hemsleyana* n-Butanol extract. A: Negative Mode B:positive mode.

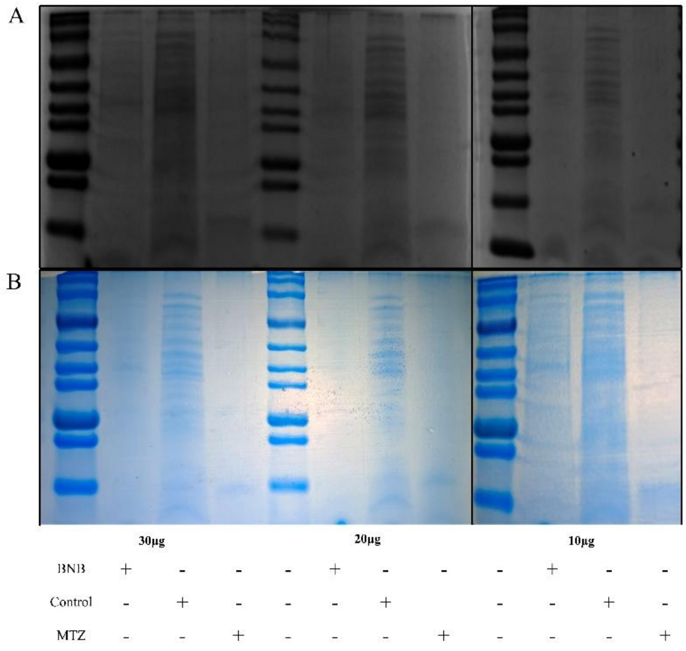

Figure S2. The intracellular protein expression of *Porphyromonas gingivalis* was as follows (the Control group consisted of P.g in the normal logarithmic growth phase, the BNB group consisted of P.g cultured at MIC concentration during the logarithmic growth phase, and the MTZ group consisted of P.g cultured with a metronidazole concentration of 500 μg/mL during the logarithmic growth phase).

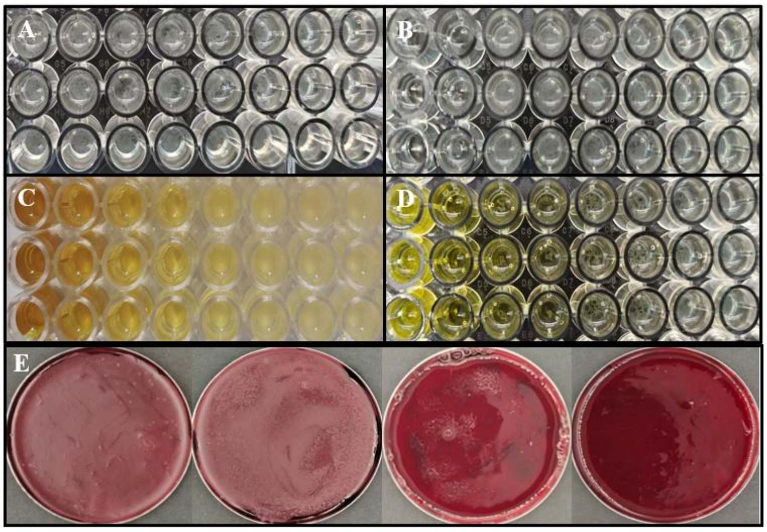

Figure S3. Determination of the Antibacterial and Bactericidal Effects of extract of compounds. (A) Acanthoside B (B)Limonin, (C) Calenduloside E, (D) Dihydroberberine, (E) Observation of Dihydroberberine's antibacterial spread on agar plates（1/2MIC，MIC，2MIC，4MIC）.

Table S1. MIC and MBC of Acanthoside B, Limonin, Calenduloside E, Dihydroberberine.

**Extraction Solvent**	**MIC（μg/mL）**	**MBC（μg/mL）**
Acanthoside B	＞500	＞500
Limonin	250	500
Calenduloside E	500	＞500
Dihydroberberine	31.25	62.50
